# State-of-the-Art: DTM Generation Using Airborne LIDAR Data

**DOI:** 10.3390/s17010150

**Published:** 2017-01-14

**Authors:** Ziyue Chen, Bingbo Gao, Bernard Devereux

**Affiliations:** 1 College of Global Change and Earth System Science, Beijing Normal University, 19 Xinjiekouwai Street, Beijing 100875, China; 2 Beijing Research Center for Information Technology in Agriculture, Beijing Academy of Agriculture and Forestry Sciences, Beijing 100097, China; gaobb@nercita.org.cn; 3 Department of Geography, University of Cambridge UK, CB2 3EN Cambridge, UK; bjd1@cam.ac.uk

**Keywords:** DTM generation, surface-based, morphology-based, TIN-based, segmentation and classification, statistical analysis, multi-scale comparison

## Abstract

Digital terrain model (DTM) generation is the fundamental application of airborne Lidar data. In past decades, a large body of studies has been conducted to present and experiment a variety of DTM generation methods. Although great progress has been made, DTM generation, especially DTM generation in specific terrain situations, remains challenging. This research introduces the general principles of DTM generation and reviews diverse mainstream DTM generation methods. In accordance with the filtering strategy, these methods are classified into six categories: surface-based adjustment; morphology-based filtering, triangulated irregular network (TIN)-based refinement, segmentation and classification, statistical analysis and multi-scale comparison. Typical methods for each category are briefly introduced and the merits and limitations of each category are discussed accordingly. Despite different categories of filtering strategies, these DTM generation methods present similar difficulties when implemented in sharply changing terrain, areas with dense non-ground features and complicated landscapes. This paper suggests that the fusion of multi-sources and integration of different methods can be effective ways for improving the performance of DTM generation.

## 1. Introduction

In past decades, the processing and applications of airborne Lidar (Light detection and ranging) data have been increasingly studied. Due to its high resolution in both horizontal and vertical directions, airborne Lidar data can be employed for monitoring the change of landscape configurations, establishing building structures, analyzing tree volumes and creating 3D urban models. Although applications of Lidar data vary, these subjects are built around one necessary procedure: the generation of digital terrain models (DTMs) using raw Lidar point clouds.

Raw Lidar point clouds include ground and non-ground points. Through interpolation, the entire point cloud can be transformed to a digital surface model (DSM) whilst the ground points can be transformed into a DTM (Although “DTM” is a frequently used term in specific papers, the term “DEM” (digital elevation model) is sometimes employed by researchers to define the surface created using ground points. This terminology strategy may be a potential risk to a broad readership. DTM refers to the bare earth surface, whereas DSM refers to a model that corresponds to the elevation of the surface of man-made or natural objects (such as building and trees) and, if no such objects exist, the bare earth. DEM is thus a more generic term that could represent DTM, DSM, or any other elevation models. With the growing use of the term “DSM” in recent Lidar studies, it is recommended to employ “DTM” for specifically describing the terrain surface generated from raw point clouds). As a key step of Lidar data processing, the quality of DTMs generated from raw point clouds not only influences the accuracy and visual effects of these models per se, but also decides the reliability of other products based on these DTMs, such as nDSM (normalized digital surface model, DSM-DTM), individual tree and building models and land cover maps. Therefore, it is of both theoretical and practical significance to propose effective algorithms for DTM generation. In the past two decades, many DTM generation algorithms have been developed. These methods aim to produce DTMs from different perspectives, such as block-minimum, slope operator, triangulated irregular network (TIN) modelling and raster calculation. Some DTM generation methods have been intendedly designed for such specific landscape types as forests or urban areas, whilst other algorithms are proposed for DTM generation in general landscape types.

To examine the performance of DTM generation methods under different circumstances, Sithole and Vosselman [1] carried out a comparative experiment to test eight classic DTM generation methods in 15 sampling sites. This study works as important reference for choosing appropriate DTM generation approaches according to specific terrain situations. In addition, this project has collected standard reference data and a variety of sample data, based on which researchers can experiment, evaluate and compare their own algorithms with existing methods. The ISPRS (International Society for Photogrammetry and Remote Sensing) sample data (http://www.itc.nl/isprswgIII-3/filtertest/index.html) has become one of the most important sources for experiments and accuracy assessment since 2003 onwards.

Although a large body of algorithms has been proposed, DTM generation is still challenging [2,3,4]. DTM generation methods are usually applied to large-scale sites. Therefore, it is very difficult to use a set of limited parameters for separating a complexity of terrain relief from a variety of non-ground features. This explains the reason why DTM generation in urban areas is particularly difficult. Sithole and Vosselman [1]’s experiment examined the suitability of mainstream DTM generation methods and promoted the research of DTM generation significantly. Inspired by previous studies, many new DTM generation methods have been proposed and examined using the ISPRS sample data in the past decade. Additionally, some scholars [5,6,7] further concluded recent development of Lidar-based DTM generation. Zhang and Men [5] concluded advantages and disadvantages of some mainstream DTM generation algorithms, and proposed five recommendations for better DTM generation: adaptive thresholds, combination of different methods, multiple sources, complex terrain filtering and advanced classification methods. However, this research did not provide detailed explanations and feasible approaches to implement the recommendations. Liu [6] reviewed recent development of Lidar systems and processing algorithms, and discussed some critical issues: Lidar data filtering, model selection, interpolation methods, DTM resolution and Lidar data reduction. Although this paper introduced several categories of DTM generation algorithms, only a small proportion of DTM generation methods were proposed since 2003. Meng et al. [7] conducted a comprehensive review of existing ground filtering algorithms. This is by far the most recent and detailed review of Lidar-based DTM generation. Slightly different from previous nomenclature, this research classified six groups of ground filters: segmentation and cluster-based filters, morphological filters, directional scanning filters, contour-based filters, TIN-based filters and interpolation-based filters. This research illustrated typical algorithms for each category, and analyzed the advantages and disadvantages for specific filters. Meng et al. [7] further examined the performance of ten mainstream ground filtering algorithms in three types of terrain: sites with rough slope and dense vegetation, sites with relatively flat urban areas containing objects of various sizes and shapes, and sites with rough terrain and discontinuous surfaces. Based on the comparison results, Meng et al. [7] suggested that ground filtering remained challenging in surfaces with rough terrain or discontinuous slope, dense forest canopies and regions with low vegetation.

With improved Lidar systems and growing availability of Lidar data, research on Lidar-based DTM generation is receiving increasing attention. Although some review papers [1,5,6,7] have reviewed a large body of ground filtering algorithms, these papers did not include the latest developments of Lidar-based DTM generation, which effectively addressed some critical issues proposed by previous studies and improved the reliability of general DTM generation. To this end, this study aims to conduct a comprehensive review of existing DTM generation methods and provides useful information for scholars to choose, implement and improve DTM generation methods. Firstly, the general principle and necessary data processing of DTM generation is introduced. Following this, existing DTM generation methods are classified into different categories and a brief explanation is given to each approach. Next, we summarize some improvement and main challenges in the subject of DTM generation, based on the understanding of existing methods. Furthermore, we propose some potential solutions to these challenges and suggest some promising directions for future studies.

## 2. General Principle of Digital Terrain Models (DTM) Generation

Although DTM generation methods vary, most of them share several main steps (albeit conducted in different orders): data pre-processing, ground point filtering and interpolation (except for TIN-based algorithms). A brief explanation of these tasks is given as follows.

### 2.1. Data Pre-Processing

Traditionally, Lidar systems can be classified as discrete Lidar and full-waveform Lidar. Different from discrete Lidar systems, which can usually record multiple echoes per emitted pulse, the full-waveform Lidar systems can record the complete waveform of backscattered signal echoes. Compared with discrete Lidar data, full-waveform Lidar data provides additional attributes, such as the neighborhood relationships between waveforms [8] and the differential laser cross-section [9,10] for classification in forests and urban areas. Although some researchers [11,12] employed full-waveform Lidar data for DTM generation, most studies simply decomposed full-waveform Lidar data into discrete Lidar point clouds and improves the accuracy of output DTMs by producing dense points. In this case, this paper mainly introduces the pre-processing of discrete Lidar data.

Pre-processing is necessary before raw Lidar point clouds can be applied to DTM generation. The most important task for Lidar data pre-processing is the removal of outliers. Due to the existence of ray multipath and Lidar system error, some points from the Lidar point cloud are of extremely low elevation. According to their elevation, there are two types of outliers: global outliers and local outliers. Global outliers have elevation values that are the lowest across the study site. The elevation values of local outliers are obviously lower than their surrounding points, but not always the lowest in the study site. Most DTM generation methods are based on morphological assumption. The lowest point in each cell is usually regarded as a ground point. If an outlier with extremely low elevation is set as a ground point, serious biases will be produced in the process of ground point filtering. Furthermore, this type of bias can hardly be corrected. In this case, effective removal of outliers is a key step for DTM generation.

The elevation difference between global outliers and local outliers requires different filtering strategy. The global outliers are the lowest points in the data set and take up a small proportion of all points. Therefore, it is comparatively easy to filter these noises. Quantile classification can be conducted and each point is classified into different groups according to its elevation value. Next, researchers can remove a small proportion of (in accordance with the quality of data sets) obvious global outliers from the raw data [13,14,15]. Thus, the point density of the data set is not reduced much and most obvious noises can be filtered.

Local outliers are much lower than neighboring points, but are not always global lowest points. As a result, simple statistical analysis cannot detect and filter local outliers. Some algorithms have been proposed for detecting local outliers. Outliers can be filtered using distribution-based approaches [13,14], mathematical morphology methods [16,17], the density-based method [18,19] and extended local-minimum method [2]. Silven-Cardenas and Wang [14] and Meng et al. [13] employed the elevation histogram to remove points with extreme elevation values and a Delaunay triangulation to examine remaining outliers. For each point, Kobler et al. [17] computed the vertical difference D between its elevation and the mean elevation of its neighbouring points. Next, all points were ranked in accordance with D. Following this, P percent of points with the largest negative D value was discarded as outliers. Chen et al. [16] assumed that outliers were scattered and meters lower than their neighbouring points. Therefore, those points that were obviously lower than their neighbouring points were deleted as outliers. Breunig et al. [18] proposed a local outlier factor (LOF) to measure the degree to which one object is isolated from its surrounding neighborhood. The object that is highly isolated from its neighboring objects is more likely to be a local outlier. By setting appropriate thresholds for the LOF value, different outliers can be efficiently detected. Those points with a large LOF value were removed as outliers. To filter outliers that may appear in various scales, Sotoodeh [19] employed global and then local outlier detection using Delaunay triangulation, Euclidean Minimum Spanning Tree (EMST) generation and Gabriel Graph (GG) generation. Chen et al. [2] analyzed the elevation of several lowest points in each cell to decide the appropriate ground point. If the difference between the elevation of the lowest point and the mean elevation of rest lowest points is smaller than a given threshold, then the lowest point can be set as the ground point. If the difference is larger than the threshold, then the lowest point is more likely an outlier and thus discarded. Then the second lowest point is examined following the same process. This iteration continues until one qualified ground point is found. 

In addition to elevation outliers, some methods employ the intensity for DTM generation. Therefore, not only elevation outliers, but also intensity outliers can lead to serious biases in the process of DTM generation. Many factors can negatively influence the intensity value of Lidar pulses. Vain and Kaasalainen [20] introduced different factors that may affect the intensity and the methodology for intensity correction. When elevation and intensity outliers have been filtered, the point cloud is ready for ground point filtering.

### 2.2. Ground Point Filtering

Filtering ground points from raw point clouds is the crucial step for DTM generation. Although a large body of studies has proposed different ground filtering algorithms, some general strategies are commonly employed. Firstly, some seed ground points are selected. As introduced above, most studies regard the lowest point within a cell as the ground point. Following this, rest points are classified as ground points and non-ground points by analyzing the spatial correlation between unclassified points and pre-set ground points. As we know, terrain surface is continuous. The closer two ground points are, the smaller their elevation difference is. In other words, closely located ground points usually have a similar elevation value whilst non-ground points may cause sudden relief change across a short horizontal distance. Following this rule, researchers can set slope (elevation difference/horizontal distance) thresholds and employ the pre-set ground points to examine the remaining unclassified points using these thresholds. If the slope between a candidate point and the seed ground point is smaller than the threshold, then the elevation difference between them is more likely to be caused by terrain relief and the candidate point is set as a ground point. If the slope is larger than the threshold, then the elevation difference is more likely to be caused by non-ground objects and this candidate point is set as a non-ground point. This rule is simplified and ideal, and has been extended and versified by many scholars. Nevertheless, the use of morphological thresholds is a fundamental strategy for ground point filtering using Lidar point clouds.

Methodologies of ground point filtering are the main focus of this paper and the introduction of specific algorithms will be presented in the following section.

### 2.3. Interpolation

DTMs may be presented as raster images or triangulated irregular network (TIN). Excepted for TIN-based DTMs, interpolation is required to transfer scattered ground points to grid-based DTMs. There are two main types of interpolation methods: the deterministic and probabilistic methods [21]. Deterministic methods regard the estimated value of un-sampled areas as the true value without any uncertainty. This type of method is effective when sampling points are densely distributed and the physical mechanic is known. When sampling points are sparsely distributed or the physical mechanic is unknown, it is inappropriate to ignore the estimation error. Inverse distance weighted (IDW), trend surface (TS) and radial basis function (RBF) are commonly used deterministic methods. IDW and TS fail to feature mountain ridges and valleys well when Lidar data is not dense enough. RBF enables researchers to retain such terrain features as mountain ridges and valleys if parameters of RBF are set properly. However, for a large study site, it is very difficult for researchers to employ unified parameters to build complicated terrain surface.

Probabilistic methods hypothesize that there is a random variable for each location and a set of fixed values for an unsampled location. When an estimated value is given to the unsampled location, the probability of occurrence can be calculated as well [21]. This type of method works efficiently when prior knowledge is missing or the point density is low. Linear prediction, Kriging and conditional simulation are typical probabilistic methods. These methods employ the variogram to estimate the missing value [22]. Through interpolation, the spatial variation of the study area is comprehensively considered. Therefore, this type of method is more likely to be generalized. Furthermore, the uncertainty feature of estimated value gives researchers significant reference on the reliability of results. However, the Kriging and linear prediction method (mathematically equivalent to Kriging method) may lead to smoothing effects and the loss of some terrain details whilst the conditional simulation may result in large estimation errors.

Although experiments have been carried out to examine the performance of different interpolation methods, Fisher and Tate [23] pointed out that there seemed to be no preferable interpolation algorithm for Lidar data for all landscapes. As a result, researchers are suggested to choose appropriate interpolation algorithms according to specific terrain situation. If a large study site is of complicated terrain relief and landscape features, researchers can divide the entire data set into small parts according to terrain characteristics and conduct interpolation respectively.

## 3. DTM Generation Methods for Different Categories

As explained above, ground point filtering is the key factor for DTM generation. In the past decades, ground point filters have been massively studied. Since each method may be understood from different perspectives, the classification of ground point filters is flexible and there are no fixed categories. Inspired by previous studies [1,5,6,7] and recent development of new methods, we classify existing ground point filters into a set of categories according to their filtering strategies. The general introduction and typical methods for each filter category are introduced as follows. 

### 3.1. Surface-Based Adjustment

The use of local minima is one of widely used ground filtering methods. This method regards the lowest point in a moving window as a ground point. By moving this window, one ground point can be selected for each cell and a DTM can thus be established using these local-minimum points. This method works effectively in flat terrain with few non-ground objects but struggles to achieve the balance between fine resolution (requiring a small grid size) and few noises (requiring a large grid size to remove large non-ground features) in such landscapes as urban areas and dense forests.

To solve this problem, researchers proposed different approaches. Amongst these methods, surface-based adjustment has become one frequently employed strategy for generating high quality DTMs. This type of method first creates an initial surface using part of the control ground points. Then, according to different error characteristics (e.g., the residual to the initial surface), qualified ground points are filtered and added to the initial surface for refining the output DTM. Through iterations, the initial surface is gradually adjusted to a fine-resolution DTM with satisfactory accuracy.

Kraus and Pfeifer [24,25] proposed a DTM generation algorithm based on robust linear prediction, which has been widely accepted by researchers. Firstly, a rough estimation of the terrain surface is computed using some control ground points (usually acquired using the lowest point for each cell). Next, the residuals (oriented distances from the surface to measured points) are calculated and each point is then given a weight according to its residual. Points with a high weight attract the surface whilst points with a low weight have limited influence on the run of the surface. The iteration continues until a stable surface is acquired or the maximum number of iteration is reached. Inspired by this algorithm, some other methods have also been presented following the principle of refining the DTM gradually [26,27,28]. Pfeifer et al. [27] and Wack and Wimmer [28] converted the original Lidar point clouds to raster images and conducted hierarchical calculation that significantly enhanced the filtering efficiency. By comparing the coarse DTM with high-resolution sources, the output DTM was improved gradually. Elmqvist [26] employed an active shape model to approximate the real terrain surface. By minimizing the energy function through iteration, this method is effective for DTM generation using very dense point clouds. Kobler et al. [17] proposed a repetitive interpolation method. This approach attempts to filter non-ground points through several steps. First, some traditional methods are employed to preliminarily remove most outliers and non-ground returns. Following this, a REIN (REpetitive INterpolation) strategy is proposed to further filter remaining non-ground points. The REIN method generates a series of TINs using randomly selected sets of Lidar points and then calculates the distribution of estimate elevation at different DTM locations. By comparing the distribution of estimate elevation with the global mean offset, remaining non-ground points can be effectively filtered. Chen et al. [29] used an iterative terrain recovery approach for DTM generation. This algorithm first registers last-return points, and then layers them by dividing Lidar points into different elevation layers. Then the detection of ground points and refinement of the output DTM is conducted from the top layer to the bottom layer. Recently, some advanced surface-filters were proposed to further improve the reliability of DTM generation. Maguya et al. [30] proposed an adaptive algorithm for large-scale DTM interpolation in steep forested terrain. Firstly, a set of local minima points were selected to produce trend surface for the following process. Different from previous studies, this method employed two different equations to simulate both linear and quadratic trend surfaces. A candidate point in the cloud was examined by the trend surface and considered as a ground (non-ground points) if the updated trend surface with this candidate point was of r^2^ larger (smaller) than the original surface. Through iteration, two DTMs generated using the linear and the quadratic models. The DTM with smaller r^2^ was discarded. If neither model achieved satisfactory result, a cubic spline model was employed to generate DTMs under steep situations. Through comprehensive use of multiple surface filters, this method proved to be effective even in some steep areas. Zhang et al. [31] brought a cloth simulation filter (CSF), which is a recent development of computer science, into DTM generation. First, the point cloud is turned upside-down, and then a cloth (originally flat) is put on the inverted surface. Next, the shape of the cloth (position of particles) was adjusted through functions that explained gravity, intersections and inner forces on the cloth. Finally, the types of cloth particles (unmovable and movable) were used to examine Lidar points in the cloud and qualified ground points were filtered. This method further employed a post-processing method to deal with sharply changing terrain. The results proved that the CSF achieved satisfactory accuracy in most terrain and the post-processing significantly enhances the performance of CSF in steep terrain. 

**Figure 1:**
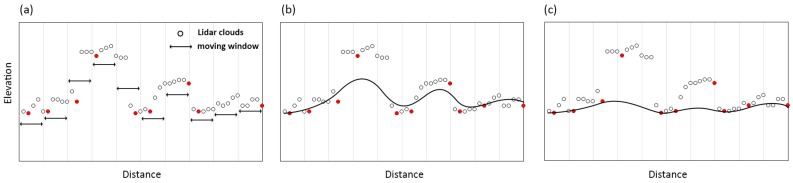
The schematic diagram of surface-based DTM generation methods. (**a**) A lowest point is selected for each cell; (**b**) A coarse surface is produced based on these pre-selected points; (**c**) A refined DTM is generated based on the residue between the coarse surface and the elevation of rest points.

A schematic diagram to explain the principle of surface-based DTM generation methods is demonstrated as Figure 1.

The surface-based filters were widely used and achieved satisfactory accuracy in most terrain situations. However, this type of method may be problematic in preserving terrain details (e.g., sharp ridges and cliffs) and tend to misclassify small non-ground objects [32].

### 3.2. Morphology-Based Filtering

In addition to surface-based adjustment, many researchers have proposed methods for generating DTMs based on morphological filtering. This type of method generally works by designing specific slope operators, which describe admissible elevation differences depending on horizontal distances. Vosselman [33] proposed a filtering approach based on mathematical morphology and retained terrain details by analyzing elevation differences among neighboring points. This method was varied by some scholars [34,35,36]. To reduce the influence of terrain relief, Sithole [34] introduced a local operator that can alter parameters as a function of the slope of the terrain, whilst Roggero [35] considered local morphology by setting the value of terrain parameters. Zakšek and Pfeifer [36] also proposed an inclined slope operator to follow the terrain. They further pointed out that elevation differences downwards are of different characteristics compared with the upwards elevation differences, which has not been discussed much by previous studies. Shao and Chen [37] proposed a “climbing and sliding” method for ground point filtering. By emulating natural movements of climbing and sliding, this method performs a local search whilst preserving advantages of a global treatment. Furthermore, some additional geometric features [38], such as erosion and dilation, as well as slope operators, have been employed to understand sloped terrain elevations. Some researchers further take into account local context for better generating DTMs. Lu et al. [3] proposed a hybrid conditional random field for automatic DTM generation. This method applies supervised learning techniques to classify ground and non-ground points and contains both discrete and continuous latent variables. For points classified as ground, the LiDAR measurements are used as an estimate of the terrain elevation. Additionally, for non-ground points, a Gaussian random field is employed for approximating the terrain elevation at these spots using nearby values.

The scan-line strategy is also an important morphology-based DTM generation method. This type of algorithm aims to detect ground points according to elevation or slope profiles [13,39]. Shan and Sampath [39] filtered urban ground points by applying a one-dimensional (1D) and bi-directional labelling filter, whereas Meng et al. [13] further extended the bi-directional filter to a multi-directional filtering method. Wang and Tseng [40] divided traditional a 1D cone-shaped slope operator into two separate operators and then employed the two operators to filter ground points respectively. The final set of grounds was the union of filtered ground points using each operator. The dual-directional profile filter (DS) filters can even be employed in both vertical and horizontal directions. The DS filter is capable of detecting sharply changing terrain, even manually made stairs, and thus suitable for urban landscapes. Hu et al. [41] employed two strategies for better removing non-ground objects without losing terrain relief. One strategy was to adapt slope operator according to terrain saliency. Through ground point segmentation, the value of terrain saliency was decided by the height difference between neighboring segments. Another strategy was to filter candidate points based on line-scanning from eight directions, instead of one direction. Since the computation involved in this method was simple, the Semi-Global Filter produced reliable DTMs with high efficiency.

Recently, growing emphasis has been given on the design of advanced morphological operators. Li et al. [42] proposed an improved Top-Hat filter for ground point filtering. This main improvement of this new Top-Hat filter was the use of additional sloped brims along with the traditional Top-Hat operator. In this case, the specific shape of this new operator worked efficiently to distinguish the elevation caused by non-ground objects (especially large buildings) and terrain relief; this operator is thus highly suitable for urban areas. Susaki [43] proposed an adaptive morphological operator to reserve local terrain details. The original plane surface for each cell was created by fitting a specific planar equation and the optimal plan surface was achieved by minimizing the root mean square error (RMSE). For each iteration, the maximum slope for the current DTM was calculated as the slope operator to filter new ground points, which were added to update the DTM. Then, the slope operator was also adapted according to the updated DTM. Through this strategy, local terrain can be reserved effectively and automatically. Pingel et al. [44] proposed a simple morphological filter (SMRF). This method works by integrating a linearly increasing window with simple slope thresholding. SMRF not only works as an independent morphological filter, but also serves as a stable foundation, based on which other advanced progressive filters may be established. Mongus and Zalik [32] employed some connected operators for better filtering ground points. First, a grid was produced to establish the connections between points. Secondly, outliers were removed using structuring elements. Following this, some connected operators, including the area of the largest contained object, the maximal roughness of the contained objects, and the level difference by which a non-ground object should be above the neighborhood in order to be recognized, were employed to filter ground points. The use of multiple thresholds at different scales enables the method to maintain large terrain relief (e.g., mountain peaks) without retaining a variety of non-ground objects. Although this method may be problematic in removing attached objects, which is a common difficulty for most algorithms, experimental results proved that this filter worked in the most challenging areas with high computational efficiency. 

**Figure 2:**
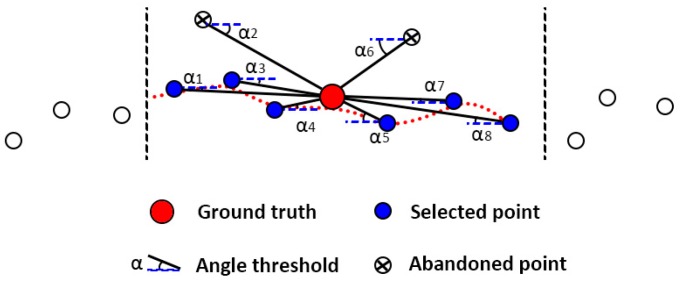
The schematic diagram of morphology-based DTM generation method. If the slope between the ground point and a candidate point is smaller than a (global or local) slope threshold, then this candidate point is set as a ground point. Otherwise, this candidate point is set as a non-ground point.

A simplified demonstration of morphology-based DTM generation method is shown as Figure 2.

Compared with surface-based filters, Mongus and Žalik [32] suggested that morphology-based filters were fairly robust for steep regions and were capable of removing small non-ground objects and preserving morphology details. Since the scale of morphological filters decides the filtering efficiency of non-ground objects with different sizes, a proper setting of the structuring element is, therefore, a major challenge for morphological filters [32]. Thus, morphology-based filtering may be challenging in terrains with a variety of non-ground objects. 

### 3.3. Triangulated Irregular Network (TIN)-Based Refinement

Another increasingly employed strategy is to generate DTMs with the help of a triangulated irregular network (TIN). In general, this type of method establishes a preliminary TIN using local minimum points, and then employs diverse examination methods to include qualified ground points and refine the TIN gradually. Axelsson [45] established a TIN with local minimum points and analyzed the relationship between residual points and the TIN. If a residual point meets certain criteria, it is included in the TIN to refine it. To avoid the edge-cutting effect, a method of mirror points is used to keep qualified edge points. Following the procedure, all ground points can be added to the final TIN. Sohn and Dowman [46] employed a “downward and upward divide-and-conquer triangulation” strategy to refine DTM iteratively. First of all, a coarse TIN surface is established using some pre-selected ground points. Then “downward divide-and-conquer” is conducted to find points lower than the trend surface and update the TIN model using these new ground points. Next, “Upward divide-and-conquer” is conducted to examine the spatial relationship between the rest of points and the TIN model. A hypothesis model is used to find candidate ground points which can be added into the TIN surface and divide local area into more planar terrain surfaces. When more than one candidate points exist for a planar terrain surface, minimum description length criterion (MDL) is used to decide the most reliable points. This iteration continues until no new ground points can be added into the TIN model. Guan el al. [47] proposed a cross-section-plane (CSP)-based filtering method. First, the entire point cloud was put into 3D grids, and multi-directional CSPs—which featured complicated 3D objects using 2D surfaces—were produced for each cell to present the DSM. Secondly, potential ground points are selected for each CSP according to the feature of elevation, intensity and multi-returns. Since this algorithm was conducted in the forested areas and laser pulses can penetrate leaves, points with single return were preliminarily set as ground points and some other potential ground points were filtered by setting judging rules (e.g., the number of neighboring ground points, elevation or intensity differences to neighboring points) based on the pre-set ground points. Next, all those potential ground points were further filtered by segmenting the scan line and selecting only the lowest point within the cell. Finally, a “merging-or-intersecting” processing is conducted to decide final ground points by merging and intersecting the sets of ground points acquired using each directional CSP, respectively.

Chen et al. [48] pointed out that the ground points on the mountain ridges were more likely to cause problems in the TIN-based methods. Classic TIN filters may sacrifice some terrain relief to avoid the inclusion of non-ground objects. Chen et al. [48] employed three strategies to solve this problem. First, the concept of ridge triangles and adjacent triangles were introduced to detect ridge ground points based on specific characteristics of the two types of triangles. Secondly, a confidence interval estimation method was employed to better select seed ground points, including both local lowest points and extracted ridge points. Finally, a simple equation was used to control the number of iterations and thus the computation efficiency has been improved significantly. Similarly, to retain terrain details around break lines, Zhang and Lin [49] combined the progressive TIN densification (PTD) with segmentation using smoothness constraint (SUSC). Firstly, original seed ground points were selected, as a key step in PTD processing. Next, regional growing method was employed based on original seed ground points and more seed ground points were filtered for the following TIN densification. With many more seed grounds, the discontinuities and terrain relief can be better reserved.

**Figure 3:**
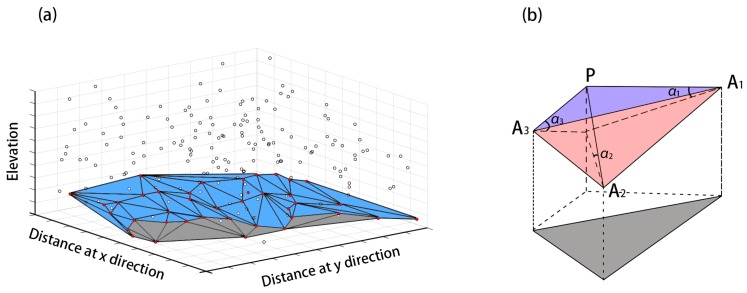
The schematic diagram of morphology-based DTM generation method. (**a**) Some of the lowest points are selected as preliminary ground points and form a coarse TIN; (**b**) Rest points are examined using triangles within this TIN model.

A schematic diagram of TIN-based DTM generation method is shown in Figure 3.

The most widely used Lidar processing software TerraScan was designed based on the Axelsson’s TIN-model [45], and the reliability and accuracy of this method has been proved by a large body of studies. However, Chen et al. [48] pointed out that progressive TIN densification (PTD) filters may have difficulties in detecting discontinuous terrains, such as sharp ridges. Thus some targeted processing should be conducted for better results. Furthermore, PTD is generally time consuming due to numerous implementations of TIN construction for a large number of points [48].

### 3.4. Segmentation and Classification

With the growing applications of airborne Lidar data in different areas, methodologies for image processing and land use (cover) classification provide important reference for DTM generation. By putting unclassified points from raw Lidar point clouds into different classes according to classification rules [50,51,52,53,54,55,56,57,58], filtered ground points can then be used for establishing DTMs. Theoretically, available features from Lidar data for land cover classification are only elevation and one band of intensity (as compared to tens, even hundreds of bands in multi-spectral remote sensing). With limited attributes, it is very difficult to conduct point-based classification using Lidar point cloud. To fill this gap, some scholars employed additional features, such as the number of returns [59,60] or the elevation difference between the first and the last return [61,62,63] to separate ground points from non-ground points. Furthermore, growing research emphasis has been given to the object-based method for DTM generation. Owing to the high resolution of Lidar data, neighbouring points are highly correlative in terms of both elevation and intensity, which makes Lidar data suitable for object-based classification [64,65]. This type of segmentation and classification-based DTM generation method is usually conducted in several steps. First, the raw point cloud is interpolated to raster images. Considering the materials for hierarchical image segmentation, not only the feature of elevation, but also features of intensity and elevation difference are converted to images. Next, image segmentation is conducted by setting some segmentation rules (e.g., segmentation scale, weight for each image layer, compactness.) and the entire image is segmented into unclassified objects. By setting classification rules for each land use (cover) type in terms of elevation, intensity, elevation difference and some geometric attributes (e.g., area, perimeter, shape index, roundness, etc.), different non-ground features can be filtered effectively and DTMs can thus be generated. Antonarakis et al. [66] employed vegetation height models, percentage canopy hit models, intensity models and skewness and kurtosis models to classify Lidar points in forest areas and bare-ground and other non-ground land cover types were separated with high accuracy. Johansen et al. [67] monitored the environmental condition of riparian zones by assessing some riparian condition indicators and efficiently classified riparian vegetation and ground by conducting object-based image analysis. Im et al. [68], Samadzadegan et al. [69] and Huang et al. [70] employed a diversity of gray-level co-occurrence Matrix (GLCM) textures (e.g., homogeneity, mean, entropy, correlation and dissimilarity) to classify trees, buildings and ground and achieved satisfactory accuracy.

Recently, some researchers proposed some new rules to improve the segmentation and classification-based filters. Niemeyer et al. [54] integrated a Random Forest classifier into a conditional random field (CRF) framework to classify grassland, road, different types of buildings, trees and low vegetation. The texture features, which have been widely used in processing remote sensing images, are also important tools for DTM generation using Lidar data. Chen et al. [71] conducted image-segmentation and then employed a shortest-distance-pair strategy, as well as a set of slope operators, to classify ground segments, instead of ground points. Thus this method is specifically suitable for removing a variety of urban objects. After image segmentation, Zhang et al. [72] selected 13 features of the geometry, radiometry, topology and echo characteristics for filtering ground segments, and the SVM tool was employed for better feature selection. Additionally, an improved connected-component labeling method, which aims to remove small and isolated segments, were employed for a more homogeneous classification results.

**Figure 4:**
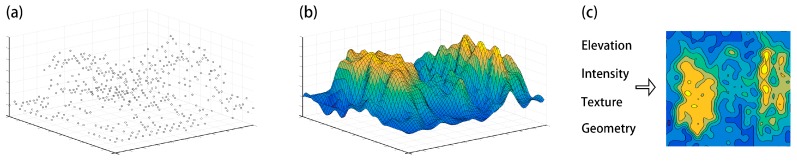
The schematic diagram of segmentation and classification-based DTM generation method. (**a**) Raw Lidar points; (**b**) Raster images produced using raw Lidar points; (**c**) After-image segmentation, unclassified segments are categorized into different land cover types by employing a diversity of features.

A schematic diagram of segmentation and classification-based DTM generation methods is shown as Figure 4.

The segmentation and classification-based methods make full use of additional geometric, texture and other features for better filtering ground points, and producing more homogeneous DTMs. This type of method can effectively remove non-ground objects of different sizes and shapes, and is thus highly suitable for urban areas. Nevertheless, Chen et al. [71] pointed out the segmentation-based DTM generation methods may struggle in densely forested areas, as scattered laser pulses that pass the gaps of tree crowns and reach the ground cannot satisfy the requirement of image segmentation. Furthermore, the reliability of this method greatly depends on the accuracy of segmentation and thus tuning segmentation parameters causes uncertainty to the performance of this type of method.

### 3.5. Statistical Analysis

In recent years, statistical analysis for DTM generation has attracted growing attention. Bartels and Wei [73] proposed an object feature extractor for terrain feature extraction. Firstly, the original Lidar point cloud was regularly gridded. Following this, the resulting matrix was decomposed and analyzed using wavelets. The result proved that detached objects could be detected in challenging hilly terrain. However, this method performs poorly in detecting large flat roofs and structures such as bridges. Bretar and Chehata [74] employed a Bayesian method for regularizing initially acquired DTM. This approach was based on the definition of an energy function that managed the refinement of a terrain surface and the process of minimizing this energy led to the final DTM. 

Some exciting progress in this field is the emerging threshold-free algorithms for DTM generation. Bartels and Wei [75] proposed an unsupervised Lidar filtering algorithm, skewness balancing. This method simply considers the skewness value of the remaining point cloud and deletes the highest point in the point cloud if the skewness value is larger than 0. As a result, very limited human interaction is required and this method is thus highly automatic. Yao et al. [76] and Bao et al. [77] also employed skewness balancing and further developed this algorithm by integrating skewness value with other classification features. Mongus and Zalik [78] proposed an unsupervised, parameter-free DTM generation method. Firstly, traditional morphological opening (with a large window size) and closing (with a small window size) was conducted repeatedly to remove lower outliers. Secondly, a series of control points to producing a pyramidal hierarchical structure for the multi-scale filtering. The selection of control points was done by employing an automatic bottom-up strategy. Hence, the selection of upper-layer control points was decided by pre-selected control points within the four neighboring cells in the lower scale. Next, thin plate spline (TPS) interpolation was conducted to produce smooth, oscillation-free trend surfaces. Finally, the ground point was filtered by analyzing the residual between the elevation of the candidate point and the trend surface. Different from previous studies, there was no pre-set threshold for the residual to filter ground points. Instead, the mean residual of all remaining points was calculated automatically and the ground point filtering was done accordingly. Additionally, the DTM resolution, which was required by multi-scale filtering, was updated automatically. As a result, this method can be highly effective and automatic. The experimental results proved this algorithm achieved high accuracy even in complicated terrains. 

**Figure 5:**
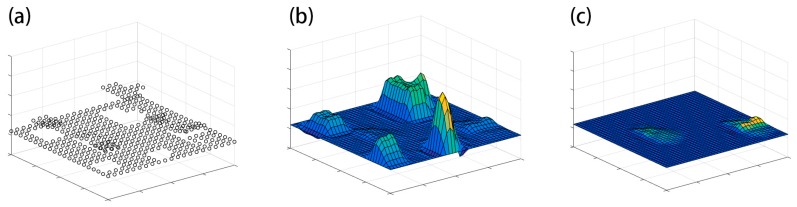
The schematic diagram of statistical analysis-based DTM generation method. (**a**) Raw Lidar points; (**b**) Global (local) high points filtered through statistical analysis; (**c**) DTM generated without filtered points.

A schematic diagram of statistical analysis-based DTM generation method is shown as Figure 5. 

Statistical analysis-based filters, especially parameter-free algorithms, reduce the uncertainty of manually tuning parameters and make the transfer of specific methods to other study sites more robust. Moreover, these methods usually perform well in generally flat terrain without complicated non-ground objects. Compared with parameter dependent filters, the reliability of this type of method may decrease significantly. Some introduced methods (e.g., [78]), achieved good accuracy in most terrains. This results from the fact that these methods do not purely rely on statistical analysis, and strategies from other ground point filters are considered as well, which is discussed more in the following section. 

### 3.6. Multi-Scale Comparison

In addition to the above discussed categories, increasing studies employ a multi-scale comparison strategy for DTM generation. This type of DTM generation method is not usually considered as an independent category. Previous studies may regard these methods as applications of surface, morphology or TIN-based methods. The main reason we separate this category from other well-accepted categories is explained as follows. Firstly, examining a point at different scales to remove noise from a range of non-ground objects is one of its theoretical differences to surface, morphological or TIN-based methods. Secondly, this type of method provides practical and reliable solutions for integrating merits of DTMs generated using different methods or parameters, which is discussed more in Section 4.

In general, this type of method works in the following steps. Several preliminary trend surfaces of different resolutions are produced. Each point in the point cloud is then examined at different scales by comparing the elevation difference between the point and different trend surfaces. If the candidate point is classified as non-ground points at a small scale (e.g., 2 m), it is assigned as a non-ground point definitely. If it is classified as a ground point at a small scale, it should be further examined at a middle scale (e.g., 10 m). This is because small non-ground objects (e.g., trees), which will be filtered at a middle scale, may be retained in the trend surface acquired at a small filtering scale. By analogy, if the point is as well classified as a ground point at a middle scale, it needs to be examined at a large scale (e.g., 50 m). This is because large non-ground objects (e.g., buildings), which will be filtered at a large scale, may be retained in the trend surface acquired at a middle filtering scale. If this point continues to be classified as a ground point at a large scale, this point is assigned as a ground point. The key issue for this type of method is the setting of a series of filtering scales. If the scale is too large, then too few ground points can be extracted and the output DTM can be over-smoothing and most terrain details are lost. On the other hand, if the scale is not large enough to filter large buildings, many building traces may be retained in the final DTM. 

Zhang et al. [79] filtered non-ground points using gradually increased window size and a slope operator that was decided automatically by comparing the filtered and unfiltered data iteratively. Chen et al. [48] employed increased window size and a building mask to iteratively update the elevation of each point in the point cloud. Li et al. [80] proposed a multi-scale mathematic morphology. First, the method extracts edge points of non-ground objects, and then processes opening operation of mathematical morphology using remaining points in the local region. Next, by comparing adjacent filter surfaces with given thresholds, surface features are extracted through increasing window size. Xiong et al. [81] designed a method for the automatic generation of DTMs. First, some regular grids are generated and non-ground points on each grid are filtered. Next, by changing (either increasing or decreasing) the grid size, new grids are produced and non-ground points retained on these grids are filtered. The iteration continues until all points have been examined and ground points have been recorded for the following DTM generation. Chen et al. [2] presented an upward-fusion DTM generation method. Different from many other methods, this method is based on raster calculation, instead of point-based filtering. First, several preliminary DTMs of different grid sizes are produced using the local minimum method. Next, upward fusion is conducted between these DTMs. This process begins with a DTM of the largest grid size and a finer-scale DTM is compared with this large-scale DTM. By setting proper thresholds, a new DTM is achieved by acquiring qualified elevation values from the finer DTM and keeping the value from the large-scale DTM when the value from the finer DTM is beyond the threshold. Iteration continues until all preliminary DTMs have been processed and a refined DTM of high resolution is generated. The experiment proved that this method produces DTMs with high accuracy. Moreover, upward fusion can be conducted using DTMs generated using different algorithms or parameters. Chen et al. [82] presented a multi-resolution hierarchical classification (MHC) algorithm for differentiating ground from non-ground points. MHC includes three levels of hierarchy, which is featured with the simultaneous increase of window size and residual threshold. For each level, the surface is iteratively approximated to the ground using thin plate spline (TPS) until no ground point is detected. Following this, these classified ground points are used to update the surface in the next iteration. Mongus et al. [83] proposed a ground point filter for urban areas. Instead of several different window sizes, this algorithm employed a function to examine a candidate point by comparing the elevation difference between this point and its neighboring points within a series of continuous window sizes (from 1 to the size of largest building size at this site). By analyzing the curve shape of this function for each point, ground points were filtered effectively. This algorithm was highly suitable for ground point filtering in urban areas with large flat-roof buildings and a diversity of non-ground objects.

Similar to Chen et al. [2]’s upward-fusion algorithm, Maguya et al. [84] first produced a series of coarse DTMs and acquired the final DTM by merging these coarse DTMs. The main differences between the two methods were that (1) these coarse DTMs were produced using the surface-based ground point filter [30], instead of the extended local-minimum method; (2) Maguya et al. [83] merged a series of coarse DTMs at the same time and the merge was conducted by considering the elevation of neighboring cells on coarse DTMs with different resolutions. On the other hand, Chen et al. [2]’s algorithms considered the comparison between neighboring cells before the process of merging and the upward-fusion was conducted simply in each corresponding cell respectively. Therefore, Maguya et al. [84]’s method may achieve better accuracy, whereas Chen et al. [2]’s method is easier to implement and can have higher fusion efficiency, which can be very convenient for improving other DTM generation methods [2]. Su et al. [85] proposed a hierarchical moving curve-fitting algorithm. The initial block size (window size) for filtering ground points was set as the size of the largest building in the study site. Then the block size was updated automatically and the filtering parameters were updated accordingly. The filtering at each scale was conducted by approximating an optimal trend surface and then each candidate point was examined by comparing the residual between its elevation and the trend surface. The trend surface was initially simulated using a second-degree polynomial function and the parameters of this function for each block was decided by the 16 lowest points in the block through the least square method. Additionally, to avoid the lack of qualified control points in each block, this algorithm employed a 25% overlapping strategy during the moving of blocks.

**Figure 6:**
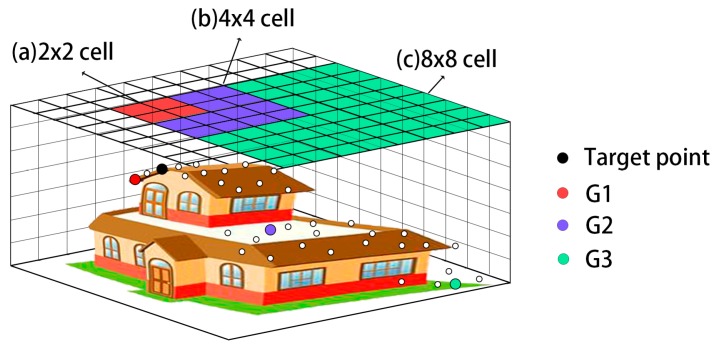
The schematic diagram of multi-scale comparison-based DTM generation method.

A schematic diagram of multi-scale comparison-based DTM generation method is shown as Figure 6.

Figure 6 is explained as follows. First, the target point is compared with the lowest point G1 in a 2 × 2 cell using a slope threshold λ1 (comparatively small). If the slope between the target point and G1 is larger than λ1, then the target point is more likely from a small non-ground object and thus set as a non-ground point. If the slope is smaller than λ1, then the target point is further compared with the lowest point G2 in a 4 × 4 cell using a slope threshold λ2 (larger than λ1). By analogy, if the slope between the target point and G2 is larger than λ2, then the target point is more likely from a middle non-ground object and set as a non-ground point. If the slope is smaller than λ2, then the target point is further compared with the lowest point G3 in an 8 × 8 cell using a slope threshold λ3 (larger than λ2). If the slope between the target point and G3 is larger than λ3, then the target point is more likely from a middle non-ground object and set as a non-ground point. If the slope is smaller than λ3, then the target point is set as a ground point.

Multi-scale comparison-based method is of special value for generating DTMs in urban areas, which are characterized by large, flat buildings and a diversity of non-ground features. Compared with other methods, the use of a large window size proves particularly effective for filtering large buildings. By adjusting the window size gradually, urban features of different sizes can be filtered effectively. However, with limited number of window sizes, this type of method may cause the loss of terrain details and sharply cut terrain relief. On the other hand, a function of automatically updating window sizes can help preserve terrain relief whilst significantly increasing computation time. In addition, this type of method is more suitable for a generally flat area and may perform poorly in rapidly changing terrain situations.

### 3.7. Overview of Different Ground Point Filters

As repeatedly stated by a large body of studies, ground point filtering from airborne Lidar data remains challenging. The main limitation is that ground point filters can have different performances under different terrains, particularly complicated terrains. Inspired by previous studies, the strengths and limitations of each type of filter are summarized in Table 1. As mentioned, the nomenclature and classification of different filtering methods employed in this study diverge from previous studies. Additionally, the understanding of terrain situations and the suitability of filters may be controversial. Thus, we generally considered the common points from a majority of scholars. Recently, some advanced ground filtering methods seem capable of solving traditional challenges in difficult terrains. However, these filters can be regarded as the combination of methods that belong to several different categories. In this section, the comparison between different categories was concluded mainly based on those methods that simply depend on one filtering strategy. Therefore, it provides some references for scholars to select proper filters according to specific terrains or propose new algorithms based on existing filters. 

## 4. Discussion

### 4.1. Recent Progress and Remaining Limitations in DTM Generation

Sithole and Vosselman [1] compared the performance of some typical DTM generation methods in a diversity of terrain situations. According to the result of accuracy assessment, algorithms proposed by Axelsson [45] and Pfeifer [27] achieved comparatively better results, as the coarse-to-fine strategy efficiently reduced large biases caused by large terrain relief. However, no method proved effective for extracting ground points in complicated terrain scenarios, which were characterized by sharply changing elevation and densely trees attached to edges. 

#### 4.1.1. DTM Generation with Improved Accuracy

Since Sithole and Vosselman [1]’s comparative experiments, many innovative methods have been proposed, which contribute to a more comprehensive and incisive understanding of ground point filtering using airborne Lidar data. First, increasing research on the design of highly automatic and generally applicable algorithms has been an exciting progress. Sithole and Vosselman [1] suggested that the human editing process took about 60%–80% of the processing time for DTM generation. As a result, these highly automatic filters, especially threshold-free algorithms [75,78], significantly enhance the time-efficiency of DTM generation and reduce possible biases caused by human editing. Another main challenge for ground point filtering is that the efficiency of most DTM generation algorithms is sensitive to specific terrains. In this case, filters that work well in forest (urban) areas may struggle in urban (forest) areas. In the past decade, some researchers [3,32,78] have proposed generally applicable ground point filters for a diversity of different terrains, which effectively reduces the time and difficulty in selecting specific DTM generation methods. Finally, based on experiments using the bench data provided by Sithole and Vosselman [1], the accuracy of DTMs generated using these new algorithms have been improved notably from previous studies.

#### 4.1.2. DTM Generation and Presentation with Improved Computational Efficiency

In addition to improved accuracy and applicability, some scholars paid special emphasis on higher computational efficiency for DTM generation. For instance, Mongus and Zalik [32] reduced the processing time for DTM generation by 98% by arranging the input Lidar data into a Max-Tree represented grid. The processing time for DTM generation and presentation is not only decided by the computational efficiency of DTM generation algorithms, but also decided by the memory storage demands for increasing in data volume. As a result, growing research emphasis has been put on the design and validation of DTM compression methods [86,87,88,89,90]. Mandlburger et al. [86] proposed an adaptive TIN refinement approach for data thinning and the experiments proved that the compression rate for DTMs generated in varying landscapes all exceeded 80%. By predicting the number of Discrete Cosine Transform (DCT) Coefficients, Forczmanski and Maleika [87] reduced the processing time for seabed DTM compression by 40%. Based on the predicted number of DCT coefficients, Forczmanski and Maleika [88] further developed a near-lossless principal component analysis (PCA)-based compression algorithm. The PCA-based compression method for generated seabed DTM demonstrated better accuracy and efficiency than traditional DCT-based methods. Meanwhile, some scholars [89,90] have worked on the better presentation of compressed DTMs in different formats or devices. Quintero et al [89] specifically designed a DTM compression algorithm for mobile devices. By replacing the full decomposition stage with obtained parameters (e.g., altitudes, contour lines and terrain roughness index) from a sub-region of the DTM, the algorithm achieved an up-to-80% compression rate. Scarmana [90] compared presented DTMs in different compression formats, including JPEG, WinZip, TIFF and PNG. Due to its high compression rate and short decoding time, the PNG (Portable Network Graphics) format was an appropriate tool for cross-platform DTM representation, storage, retrieval and display.

#### 4.1.3. Remaining Challenges for DTM Generation

In spite of many new methods for ground point filtering, DTM generation remains challenging. As explained above, each category of methods has its own strengths and limitations. However, few methods are applicable to all terrain situations, which include densely forested areas, urban areas, sharp ridges and discontinuous terrains and so forth. Furthermore, it is still difficult to employ one single filter for DTM generation in very complicated or highly fragmented terrain situations. In recent years, airborne Lidar has been increasingly applied to oceanography and hydrology [91]. However, due to the absorption and scattering effects of water on laser pulses, DTM generation in coastal regions has extra difficulties. Mohammadzadeh and Valadan Zoej [91] concluded some common challenges for DTM generation in ocean and hydrography, including dune and tidal flat measurement and coastal change and erosion.

### 4.2. Promising Directions

According to limitations in current research of DTM generation, especially ground point filtering, more emphasis should be given on two directions:

#### 4.2.1. Advanced DTM Generation by Combining Different Methods

This paper introduced different categories of DTM generation methods, and each type of method is more suitable for a certain terrains]. If the merits of different methods can be combined, the accuracy of generated DTMs can be improved significantly. As explained, some new ground point filters can be generally applied to a variety of terrains and achieve better accuracy than previous algorithms. The main improvement was that these filters, although classified into specific categories in this paper, actually combined different filter strategies. Su et al. [85]’s method successfully combined the surface-based filter and the multi-scale comparison algorithm for better removing large buildings, as well as preserving terrain details. Mongus and Zalik [32] proposed a DTM generation method, which combined the strategy of multi-scale comparison, segmentation and morphological operators, and achieved high accuracy and computational accuracy. Zhang and Lin [49] combined TIN-based and segmentation-based algorithms for a new filter, which worked effectively both in steep terrains and terrains with a variety of different objects. Mongus et al. [83] combined the strategy of morphological operators, multi-scale comparison and surface-based adjustment and effectively classified ground points from buildings of a diversity of structures. The DTM generation method proposed by Mongus and Zalik [78] has been frequently used not only because of its parameter-free strategy, but also due to its general suitability for various terrains. The efficiency of this method also mainly depends on the successful combination of the surface-based adjustment, multi-scale comparison and statistical analysis.

In addition to these individual methods that combine different filtering strategies, some scholars even proposed methods that can directly make improvements on the outputs by merging DTMs generated using different methods (software) or parameters, which is a promising tool for significantly improving DTM generation based on existing methods. Chen et al. [2] proposed an upward fusion-based method for DTM generation. This method is a raster-based method, so raster calculation can be conducted using raster DTMs generated using any methods or parameters. Therefore, this method not only works alone to generate high quality DTMs, but also serves as an efficient tool for fusing DTMs generated using different methods. For instance, when upward-fusion was conducted between DTMs generated using lasground and TerraScan, five out of nine points with large bias were filtered and the mean bias of updated DTM was improved from 0.254 m to 0.114 m. When upward-fusion was conducted between DTMs generated using TIFFS with different parameter values, 16 out of 24 points with large bias were filtered and the mean bias of the updated DTM was improved from 0.755 m to 0.206 m.

By designing specific methods combined with different filtering strategies or fusing output DTMs generated using different methods, the merits of these algorithms can be retained whilst their limitations can be offset effectively. In future studies, researchers should continue to work on more approaches for combining different ground filtering methods. In addition, it is of practical significance to examine which methods combined together may achieve the optimum results.

#### 4.2.2. Advanced DTM Generation Using Multi-Sources

Although great progress has been made in the design of new DTM generation methods and some challenging issues have been addressed to some extent, some limitations remain. Specifically, it is difficult for traditional methods to precisely generate DTMs in complicated terrain situations that are characterized with sharp terrain relief and a diversity of non-ground features. Due to its rapidly changing elevation, sharp terrain relief is very likely to be considered as non-ground objects according to morphological filters, slope operators or statistical functions. Bringing in additional features to assist the process of ground point filtering is a practical solution to this problem. Although some scholars conduct image segmentation and classification and aim to classify non-ground objects using the intensity feature, the single band of intensity attribute cannot support the task of classifying a diversity of non-ground features. Moreover, as explained, the intensity feature provided by Lidar data is not fully reliable for classification. Furthermore, the existence of discontinuity and highly fragmented landscapes inevitably prevents a satisfactory DTM generation result using airborne Lidar data solely, especially in large study sites with complicated terrain and object conditions.

In recent years, the integration of Lidar data and other data sources for land use (cover) classification has gained gpopularity. In addition, it is widely accepted that integrating Lidar data with multi-spectral remote sensing data achieves much better classification accuracy than the sole use of airborne Lidar. As a result, Lidar data has been fused with multi-spectral images [92,93,94], high-resolution imagery [95,96,97,98], airborne photography [99] and hyperspectral imagery [100,101,102] for land cover classification. Amongst these studies, most scholars considered the elevation feature from Lidar data for better classification accuracy, yet few studies employed additional data sources for better DTM generation. This imbalance may result from people’s biased understanding of DTM generation and airborne Lidar data. Many scholars believe that the main application of airborne Lidar data is DTM generation, and the sole use of airborne Lidar is more than enough for DTM generation. In fact, including additional spectral features provided by other data sources is probably a better solution for DTM generation in complicated terrains than designing advanced DTM methods using Lidar data only. Recently, an exciting development that some scholars started to apply is multiple sources for better DTM generation. Kim et al. employed the spectral feature from aerial images [103] and high-resolution satellite images [104] to better classify bare ground and building roofs, which is one common challenge in urban DTM generation. Nordbo et al. [105] employed existing building mask (geographical information on the coastline and building edges) to assist the generation of large-scale urban DTMs using airborne Lidar data. Debella-Gilo [106] combined high-resolution photogrammetric point clouds (similar to Lidar point clouds) with existing low-resolution DTMs, which worked as reference trend surface, to produce high-resolution DTMs. Instead of direct use of spectral information, Saeidi et al. [107] extracted the NDVI (Normalized Difference Vegetation Index) feature to assist the classification of bare ground, trees attached to steep terrains and large buildings in complicated landscapes and produced high-quality DTMs. Since the Laser pulses can be absorbed when hitting the water surface, geomorphic analysis at coastal regions using Lidar data solely is difficult. Therefore, the use of both airborne Lidar and imaging sensors is highly recommended [91,108]. Moretto et al. [109] fused Lidar, colour bathymetry and dgps surveys to better feature coastal line terrain, which is a typical demonstration of employing multiple sources to generate DTMs in complicated terrains. However, the implementation of multi-source supported DTM generation should be further explored through many more case studies conducted in complicated and highly fragmented landscapes with steep and discontinuous terrains and a variety of attached objects.

Full-waveform Lidar data has been increasingly examined, as it provides additional spatial correlation and useful features. Mallet et al. [9,10] classified ground, vegetation and building points using such specific features as differential laser cross-section from full-waveform Lidar data, whilst Jutzi and Stilla [8] conducted urban land cover classification using the neighborhood relationships between waveforms. Considering that full-waveform Lidar data can provide many additional features, this data source is of great potential for DTM generation in very complicated or highly fragmented terrain situations, which requires a diversity of features to distinguish steep terrain from non-ground objects.

## 5. Summary

Over the past few decades, methods for digital terrain model (DTM) generation have been intensively studied and many algorithms have been proposed to derive DTMs under different terrain situations. This paper reviews recent progress of DTM generation and categorized existing DTM generation methods into six major classes: surface-based adjustment, morphology-based filtering, TIN-based refinement, segmentation and classification, statistical analysis and multi-scale comparison. The principle, typical algorithms, suitability and limitations for each category are explained and compared. 

Since DTM generation methods of different categories have their own strengths and limitations in specific terrains, the simple use of one type of ground point filter can hardly satisfy requirements of a diversity of terrains. To this end, some new ground point filters have been proposed recently to make generally applicable DTM generation possible. These algorithms have successfully combined several categories of ground point filters, and can thus retain terrain details and remove a diversity of objects. Therefore, these new methods proved their capability of producing reliable DTMs even under many challenging terrains, indicating a significant progress in the principle and implementations of DTM generation.

Despite the development in the reliability and generalization of DTM generation, some limitations still exist. Due to limited features provided by light detection and ranging (Lidar) data, it remains very difficult, if not impossible, to simultaneously distinguish complicated terrain situations (e.g., discontinuities and shape ridges), highly fragmented landscapes, and a variety of objects using airborne Lidar data solely. This challenge is inevitable in DTM generation implementation conducted in large areas, which include a variety of complicated terrain relief and non-ground objects. An effective solution is to integrate additional sources with airborne Lidar data for better DTM generation. Although the fusion of multi-spectral images with Lidar data has been frequently implemented in land cover/use classification and other fields, most scholars only considered the elevation feature from Lidar data for better classification whilst few studies employed additional sources for better DTM generation. Several recent studies have started to combine Lidar data with additional sources for generating DTMs in complicated terrains, which should be a promising direction for further development. The selection of data sources, the method for data fusion, and the suitability of terrains are the key issues for multi-source supported DTM generation, which should be explored through many more case studies.

This review presented recent developments, remaining challenges and promising directions for DTM generation using airborne Lidar data. It also provides useful references for scholars to properly choose methods according to specific terrains, or design new methods based on a better understanding of existing algorithms.

## Figures and Tables

**Table 1:** Characteristics of different ground point filters.

Filtering Methods	Suitable for	Not Suitable for	Memory Storage Demands	Computational Efficiency ^2^
Surface-based	Forested areas	Rough and steep terrains	High	Middle
Morphology-based	Steepterrains ^1^, Terrains with small objects	Terrains with various objects	Low	High
TIN-based	Steep terrains	Urban areas, Discontinuous terrains	Middle	Middle
Segmentation-based	Urban areas, Terrains with various objects	Rough and steep terrains, Dense forests	NA ^3^	NA ^4^
Statistical analysis	Generally flat terrains	Terrains with various objects	Low	Low
Multi-scale comparison	Urban areas	Rough and steep terrains	Middle	Low

^1^ The suitability of morphology-based filters in steep terrains is controversial. Although some scholars claimed this type of filter struggles in steep terrains, Mongus and Žalik [32] pointed out that morphological filters worked fairly well in steep terrains. Due to this controversy, it is suggested that the morphological filters should be employed in steep terrains without a diversity of objects, which would otherwise significantly reduce the reliability of generated DTMs; ^2^ The computational efficiency of different categories is generalized according to previous experiments [48,49]. Thus, the conclusions made here purely depend on the performance of some typical algorithms for each category and the time efficiency for specific algorithms can be different; ^3^ The memory storage demands for the segmentation-based method varies significantly with the resolution of interpolated DSMs and image segmentation scale and thus cannot be compared with other filters; ^4^ The computational efficiency for the segmentation-based method highly depends on some necessary manual parameter settings during image-segmentation and classification, and can hardly be compared with other highly automatic methods.
